# Effects of a 6 week core strengthening training on measures of physical and athletic performance in adolescent male sub-elite handball players

**DOI:** 10.3389/fspor.2022.1037078

**Published:** 2022-11-07

**Authors:** Julian Bauer, Thomas Muehlbauer

**Affiliations:** ^1^Division of Movement and Training Sciences/Biomechanics of Sport, University of Duisburg-Essen, Essen, Germany; ^2^Department of Sport Science, Human Performance Research Centre, University of Konstanz, Konstanz, Germany

**Keywords:** trunk exercises, strength endurance, shoulder mobility/stability, throwing velocity, youth

## Abstract

The objective was to investigate the effects of a 6-week core strengthening training within the regular handball training sessions compared to regular handball training only. Male sub-elite handball players were randomly assigned to an intervention (INT: *n* = 13; age: 16.9 ± 0.6 years) or a control (CON: *n* = 13; age: 17.2 ± 0.8 years) group. The INT group performed the “big 3” core exercises *cross curls-up, side bridge* (both sides), and the *quadrupedal stance* (“birddog exercise”) triweekly for 20–30 min while the CON group conducted regular handball training only. Pre- and post-training assessments included measures of muscular endurance (Closed Kinetic Chain Upper Extremity Stability Test [CKCUEST] and the Bourban test), shoulder mobility/stability (Upper Quarter Y Balance [YBT-UQ] test), and throwing velocity. The ANCOVA revealed significant differences between means in favour of the INT group for the dorsal chain (*p* < 0.001, $\eta_{\text{p}}^{2}$ = 0.46) and the lateral chain (left side: *p* = 0.015, $\eta_{\text{p}}^{2}$ = 0.22; right side: *p* = 0.039, $\eta_{\text{p}}^{2}$ = 0.17) of the Bourban test, the composite score (*p* = 0.024, $\eta_{\text{p}}^{2}$ = 0.20) of the throwing arm reach and the inferolateral reach direction (*p* = 0.038, $\eta_{\text{p}}^{2}$ = 0.17), and the composite score (*p* = 0.027, $\eta_{\text{p}}^{2}$ = 0.19) of the non-throwing arm reach of the YBT-UQ. However, performance in the CKCUEST and throwing velocity did not show any group-specific changes. Therefore, 6 weeks of core strengthening training were effective in improving some components of physical but no handball-specific athletic (i.e., throwing velocity) performance in adolescent male sub-elite handball players. Practitioners may still opt for this training regimen when stimulus variability is sought or when a low load/low movement approach (e.g., during rehabilitation) is favoured.

## Introduction

Behm et al. (1) defined the core as “the axial skeleton (which includes the pelvic girdle and shoulder girdles) and all soft tissues (i.e., articular and fibro-cartilage, ligaments, tendons, muscles, and fascia) with a proximal attachment originating on the axial skeleton, regardless of whether the soft tissue terminates on the axial or appendicular skeleton (upper and lower extremities)” [2, p. 92]. Based on this definition, the core represents an integral part to transfer and control forces in the kinetic chain during every movement (2). Still, the precise definition of what the core, sometimes referred to as trunk, is, remains unequivocal, and definitions vary depending on whether being more on a rehabilitation or athletic conditioning perspective (1).

The core plays an important role in handball during the most important technique of throwing (3, 4). During throws, the transfer of force has been reported to go through the centre of the body in a proximal to distal sequencing which requires an effective kinetic chain and a well-developed core (5). Greater throwing velocities are documented in successful throws (6), while to achieve these, the core plays a major role (7–9). In addition, throwing velocity is also influenced by well-developed shoulder mobility and stability (10) as a wide elbow extension and shoulder internal rotation together with a maximal external rotation (7) facilitate the acceleration of the ball. In this context, elite handball players have also been reported to possess higher athletic performance in terms of core strength and power than amateur players (3, 11). The core functions as a kinetic link enable dynamic activities of the extremities (12) and play a vital role in the prevention of injuries (13) already in young players as the handball-specific exposure already starts at that age. Therefore, core strengthening training should already be part of youth and adolescent handball training routines to improve the important key performance aspect of throwing velocity.

Several intervention studies examined the effectiveness of core strengthening training on throwing velocity and athletic performance with age being a potential subject-related moderator variable. In this line of thought, Saeterbakken et al. (12) reported that between-group differences in their sub-group analyses yielded minor effects for age, but not expertise level. Therefore, classifying the following studies in relation to the factor age seems necessary. Regarding adult cohort studies, Kuhn et al. (14) investigated the effects of a 6-week in-season core stability training on increasingly unstable conditions twice a week in addition to regular handball training on maximal throwing velocity, isometric strength, and muscular endurance in 20 female handball players (age: 23.4 ± 4.4 years). The authors concluded that the intervention effectively increased isometric strength and muscular endurance as assessed through the Swiss Olympic Medical Center core performance test battery. More precisely, core endurance significantly improved in the left (23%) and right (30%) lateral core muscle chains. However, for the ventral and dorsal core muscle chains there were no significant improvements in either group. Additionally, throwing velocity did not increase in both the core stability training and the control group. Further, Dahl and van den Tillaar (15) assessed the effect of an 8-week sling-based training with rotational core exercises in contrast to plyometrics/sprint training on throwing velocity in female handball players (*n* = 25; age: 19.5 ± 2.0 years). The participants were either included in a sling-based training group or a control group. Following the training, significant group effects over time for the 7-m standing throw (3.3%), the run-up shot (1.9%), and the jump shot (2.8%) in favour of the sling-based training group were present. Additionally, the authors assessed maximal rotational velocity with a linear encoder and calculated the 1RM based on the four resistance levels of 5, 10, 15, and 20 kg. However, no significant differences were found between groups for the calculated 1RM or any other maximal rotational velocities. Moreover, Manchado et al. (16) assessed the impact of 7 core exercises for 10 weeks triweekly during the normal training sessions in 30 male junior handball players (age: 18.7 ± 3.8 years). Following the intervention, the experimental group showed a significant 4.3% improvement in the sum of different throws and the 7-m throw while the control group showed no improvements in the standing throw, the throw with run-up, or the jump throw.

Concerning adolescent cohort studies, Saeterbakken et al. (17) executed a study with 24 female high-school handball players (age: 16.6 ± 0.3 years) which were initially divided into a sling exercise training group that executed core exercises with a sling in addition to the regular handball training and a control group. Following 6 weeks of twice-a-week progressive sling core stability training, the maximal throwing velocity as assessed by a 7-m penalty throw significantly increased by 4.9% in the sling core training group while in the control group it did not. In addition, Ozmen et al. (18) conducted a study in which 20 male adolescent handball players (age: 14.90 ± 0.44 years) were randomly divided into either a core strength training group or a control group. The intervention group performed twice-weekly core strength training in addition to handball training with progressively increasing difficulty. Following the 6-week intervention, no significant changes in throwing velocity were present in either group. The authors additionally assessed dynamic balance of the lower extremities based on the star excursion balance test and tested the vertical jump height. Significant differences were reported for the anterior (12%) and posteromedial (8%) reach directions only in the core strength training group. Additionally, there were significant improvements in vertical jump height compared to pretest scores in both the core strength training group (5%) and the control group (11%). However, differences in all parameters when both, the core strength training and the control group, were compared remained nonsignificant.

In sum, the present findings on the effects of core strengthening training on throwing velocity in adolescent handball players are inconsistent in terms of results and vary with respect to the applied methodological approaches. All aforementioned interventions were executed in addition to the regular handball training sessions making it unclear whether possible effects can be attributed to the core strengthening training or a higher training volume in general. Therefore, the aim of this study is to investigate the effects of a progressive triweekly 6-week core strengthening training during handball training compared to regular handball training only on muscular endurance, shoulder mobility/stability, and handball-specific athletic performance (i.e., throwing velocity) in adolescent male sub-elite handball players. We hypothesised that both groups will improve their physical and athletic performance with superior effects for the core strengthening training group.

## Methods

### Participants

**Figure 1:**
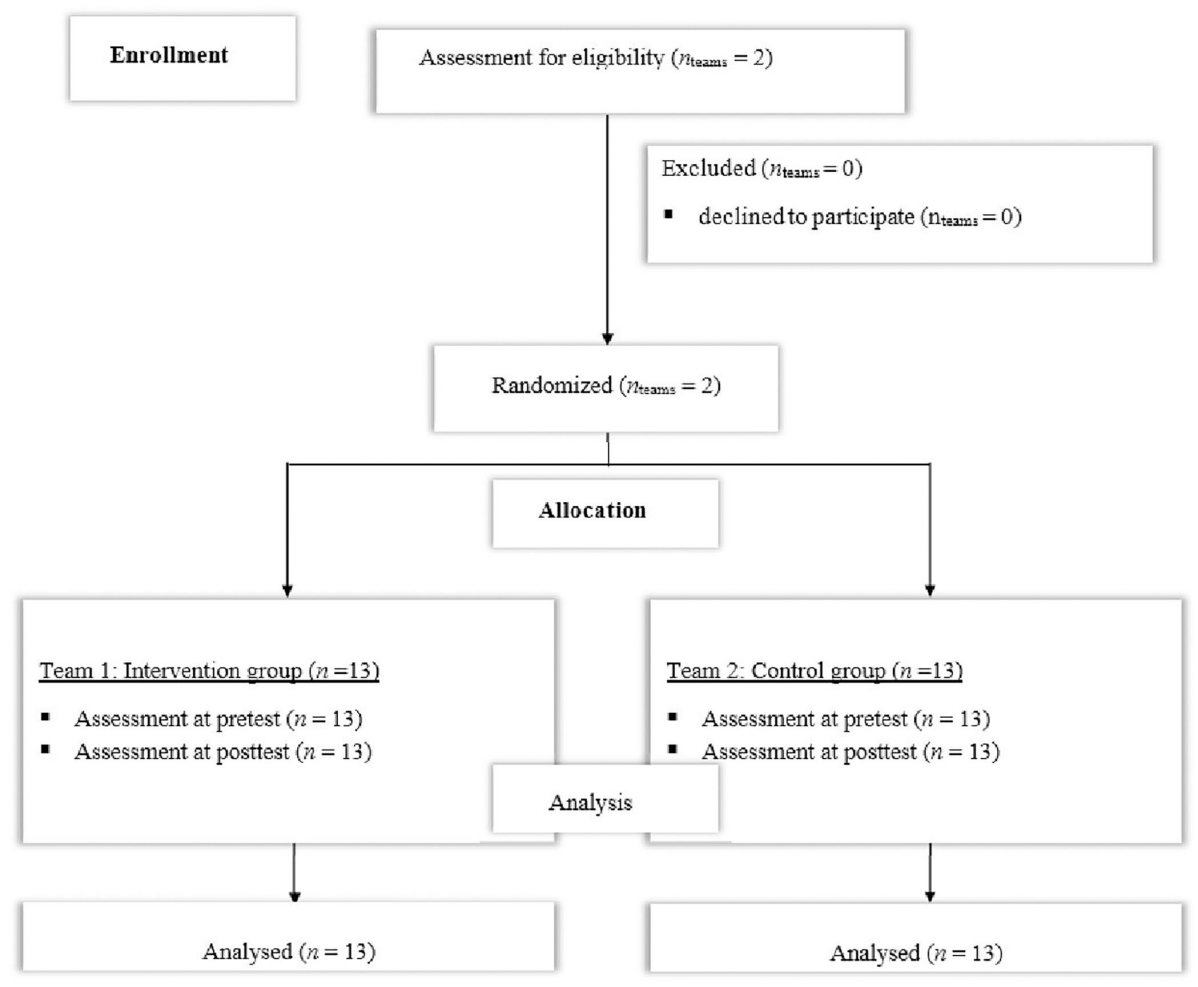
Flowchart of the progress through the phases of the study according to the CONSORT Statement 2010.

Using G^*^Power (19), a power analysis (*f* = 0.25, α = 0.05, 1-β = 0.80, number of groups: *n* = 2, number of measurements: *n* = 2, correlation among repeated measures: *r* = 0.70, drop-out rate: 10% due to reasons not attributable to the intervention) was conducted based on the medium-sized effects reported by Dahl and van den Tillaar (15) and revealed that a total sample size of *N* = 24 participants (i.e., *n* = 12 per group) would be sufficient to detect statistically significant test × group interactions. *Two* male under-19 teams competing in the same regional sub-elite playing class with comparable training regimen (three times a week for 90 min each training session and one game at the weekends) were contacted, informed about the program, and asked for their willingness to participate. As both teams were willing to participate, Research Randomizer software (www.randomizer.org) was used to allocate the teams either into the intervention (INT) or the control (CON) group (see Figure 1). All players gave written consent, and the subjects who were under the age of 18 years additionally handed in an informed consent from their parents or legal guardians. Both teams consisted of 13 participants (see Table 1) which executed both the pre- and posttest assessments. Exclusion criteria were any injuries or illnesses that were judged to may have an influence on one of the outcome parameters. Additionally, subjects were excluded when an illness or injury hindered them from training or games 2 weeks before testing. The study was carried out according to the Declaration of Helsinki (20), and the Human Ethics Committee at the University of Duisburg-Essen, Faculty of Social Sciences, approved the study protocol (TM_23.03.2020).

**Table 1:** Characteristics of the study participants (*N* = 26) by group.

**Characteristic**	**INT-group (*n =* 13)**	**CON-group (*n =* l3)**	***p*-value**
Age [yrs]	16.9 ± 0.6	17.2 ± 0.8	0.301
Maturity offset (years from PHV)	3.0 ± 0.7	3.4 ± 0.7	0.091
Body mass [kg]	81.9 ± 12.0	75.7 ± 15.6	0.269
Body height [cm]	179.1 ± 6.0	183.6 ± 5.8	0.063
BMI [kg/m^2^]	25.6 ± 4.4	22.3 ± 3.7	0.053
Arm dominance [l/r]	3/10	1/12	
Throwing arm [l/r]	3/10	1/12	
Throwing arm length [cm]	91.5 ± 3.4	93.1 ± 3.8	0.276
Non-throwing arm length [cm]	91.2 ± 3.3	93.0 ± 3.6	0.207
Training experience [yrs]	8.4 ± 3.2	8.8 ± 3.5	0.729

Data are group mean values ± standard deviations. BMI, body mass index; CON, control group; INT= intervention group; 1, left; PHV, peak height velocity; r, right.

### Core strengthening training program

The present intervention focused on a floor-based core strengthening training. The program consisted of the “big 3” core exercises *cross curl-ups*, the *side bridge* (both sides), and the *quadrupedal stance* (“birddog exercise”) as proposed by McGill (21) and Granacher et al. (22). Both the participants of the INT and the CON group performed their regular handball training routine (three times per week, 90 min per session) throughout the duration of the intervention. While the participants of the INT group executed the 6-week core strengthening training program (3 times per week; 20–30 min per session) within the first 20–30 min of their regular 90-min handball training session, the CON group spend this time with handball-specific training following the guidelines of the German Handball Association (23) which consisted of passing and throwing exercises resulting in the same temporary training load of 90 min per session for both groups. Before the first training session, one member of the testing staff demonstrated the exercises to the players, and additionally, all coaches of the INT group were given pictures of the according exercises together with detailed explanations. All exercises were executed in a non-fatigued but warmed-up condition. Weekly phone calls between the examiner of the study and the coaches were conducted to keep the handball training intensity on a similar level and to assure the compliance with the training program. All training sessions took place midst the competition period. To assure progression, the training intensity was increased every week by the change from static to dynamic movements and an increase in time from 45 s over 60 s to 75 s every 2 weeks as proposed by Granacher et al. (22) and Kuhn et al. (14) (see Table 2).

**Table 2:** Description of the core strengthening training program.

**Exercise**	**Week 1**	**Week 2**	**Week 3**	**Week 4**	**Week 5**	**Week 6**
Cross curl-ups	3 x 45 s	3 x 45 s 60 bpm	3 x 60 s	3 x 60 s 60 bpm	3 x 75 s	3 x 75 s 60 bpm
Side bridge (both sides)	3 x 45 s	3 x 45 s 60 bpm	3 x 60 s	3 x 60 s 60 bpm	3 x 75 s	3 x 75 s 60 bpm
Quadrupedal stance (bird dog exercise)	3 x 45 s	3 x 45 s 60 bpm	3 x 60 s	3 x 60 s 60 bpm	3 x 75 s	3 x 75 s 60 bpm

Weeks 1, 3, and 5 represent a static and weeks 2, 4, and 6 a dynamic (at 60 beats per minute) exercise execution. Bpm, beats per minute.

### Basic exercise position and execution of the cross curl-ups

The subjects adopted a supine position with their hands folded in the neck and their elbows pointed to the sides. The feet were put on a fitness map while their knees remained in a flexed position. In the following, the subjects curled-up until the scapula left the fitness mat (22). The subjects rotated to the left and the right alternately at a 60 bpm speed being assured by a metronome during the dynamic execution. In the static execution of the exercise, the participants only held this position and changed the upper shoulder being rotated to the front halftime of the duration, which was verbally indicated by the coaches (i.e., after 22.5 s in week 1, 30 s in week 3, and 37.5 s in week 5).

### Basic exercise position and execution of the side bridge

The subjects raised their hips until being in a straight line from the knees up to the shoulders while lying in a side position with their knees flexed. The supporting shoulder was positioned superior to the respective elbow while the other arm was held akimbo (22). During the static execution, the subjects held this position, while in the dynamic regimen, the subjects continuously raised and lowered their hips at a 60 bpm speed. The side bridge was executed for the respective duration and mode (static or dynamic) on both sides.

### Basic exercise position and execution of the quadrupedal stance

Starting from a quadrupedal stance with both hands and knees flat to the surface, the subjects lifted one leg and the contralateral arm into a horizontal position. The subjects alternately lifted and lowered their leg and contralateral arm at a 60 bpm speed during the dynamic execution while during the static mode one leg and the contralateral arm were held statically in a horizontal position with the respective sides being changed after half of the duration, which was verbally indicated by the coaches (i.e., after 22.5 s in week 1, 30 s in week 3, and 37.5 s in week 5).

## Testing procedures

### Assessment of playing and training experience

To assess training experience, each subject was asked for how many years they had been training and playing handball in a club. Additionally, the subjects were asked which arm their dominant and throwing arm is.

### Assessment of anthropometric variables

Prior to the first testing, the anthropometric variables body height, body mass, and upper limb length were assessed. From the seventh cervical spinous process (C7), upper limb measurement was carried out to the distal tip of the middle finger with the shoulder being in 90 degree abduction (24). Body height was measured with a Seca 217 (Seca, Basel, Switzerland) linear measurement scale with the subjects standing straight without shoes to the nearest 0.1 cm. Body mass was also assessed without shoes, in light sportswear that was subsequently worn during the testing, with a Seca 803 (Seca, Basel, Switzerland) electronic scale to the nearest 100 g. The body mass index (BMI) was calculated as body mass divided by the measured body height squared (kg/m^2^). Further, participants' maturity status was calculated as years from peak height velocity by using the equation provided by Moore et al. (25): maturity offset = −7.999994 + {0.0036124 × (age [yrs] × body height [cm])}. Positive values indicate that individuals have already passed their maximal growth rate.

### Assessment of muscular endurance

Muscular endurance was tested using the closed kinetic chain upper extremity stability test (CKCUEST) and the Bourban test (26) which was applied to assess core stability in three motion planes (ventral, dorsal, and lateral). Both the ventral and lateral chain postures were performed on a fitness mat while the dorsal chain was tested on a long box.

### Bourban test: Ventral chain

In the starting position of the ventral chain, the subjects placed themselves in a prone bridge with their legs straightened, face down in a vertical upper arm and parallel under-arm position with their thumbs being upright (27). From the lateral malleolus to the trochanter major, there was a straight line up to the glenohumeral joint and the greater trochanter. An adjustable alignment device with a stable vertical pole and two vertically adjustable horizontal rods (26) was moved into contact with the subject's lower back at the level of the iliac crests and then fixed in this position (22). Out of this position, the subjects were asked to alternately lift their feet about 2–5 cm from the floor based on the beat of a metronome (60 bpm – 1-s lifting, 1-s lowering). The test was stopped, and the maximum number of seconds was noted down on the scoring sheet as soon as the subjects either lost contact to the bar for longer than 1 s, could not keep up with the pace of the metronome, or could not hold up the straight position. Based on the recommendations regarding absolute reliability (28), the ventral chain test can be classified as reliable with a coefficient of variation (CoV) of 14.1% (29).

### Bourban test: Dorsal chain

The test of the dorsal chain started with the subjects laying prone on a box while not being supported at the trunk (from the upper border of the iliac crest) (22). The subjects had to hold their arms across the chest with their hands rested on the shoulders. The legs were fully extended while their feet were firmly fixed in wall bars behind the box at the wall (22). A mechanical goniometer was used to control the horizontal positioning during the whole execution. The upper horizontal reference rod of the alignment device was fixed at the level of a thoracic spinal process (22). Thereafter, the subject lowered the trunk by 30° which was again controlled for by a mechanical goniometer until the lower horizontal reference rod of the alignment device was touched at the height of the sternal angle. Based on the beat of the metronome (60 bpm – 1-s lowering, 1-s lifting the trunk), the participants continuously raised and lowered their trunk. The test was stopped, once the subjects failed to touch the upper or lower horizontal rod for two consecutive times or a total number of three times. The best trial (maximum number of seconds) was used for further analysis. Based on the recommendations regarding absolute reliability (28), the dorsal chain test can be classified as reliable with a CoV of 11.7% (29).

### Bourban test: Lateral chain

The test of the lateral chain started in a bridge position with the legs being extended and the upper foot placed on top of the lower foot. Additionally, the supporting shoulder was held superior to the respective elbow (22). While the supporting forearm was placed flat on the floor, the other arm was held akimbo. Subjects had to raise their hips until a straight line was reached from the ankles up to the shoulders. During the side bridge position, the lower horizontal reference rod of the alignment device was fixed at the height of the superior iliac crest (22). The subjects were asked to continuously raise and lower their hips based on the beat of a metronome (60 bpm – 1-s lifting, 1-s lowering). It was prohibited to lower the body touching the floor during the lowering phase. Warnings were given when the subjects did not touch the horizontal rod in the lifting phase. The test was stopped as soon as the subjects did not fulfil their task for two consecutive times or a total number of three times. After stoppage, the maximum number of seconds was noted on the scoring sheet for further analysis. The test for the lateral chain was performed for both sides with the other side being tested after a short break. Based on the recommendations regarding absolute reliability (28), the lateral chain test can be classified as reliable with a CoV of 14.6% (29).

### Closed kinetic chain upper extremity stability test

During the CKCUEST, the subjects were asked to position themselves in a push-up position with their hands placed 36 inches (~91.44 cm) apart. Their back had to be kept flat and their shoulders perpendicular to the wrists (30). Following the starting position, the subjects were requested to alternately touch the supporting hand with the mobile hand being lifted as often as possible during three 15 s trials, separated by 45-s rest period. During the 45-s rest period, the participants were allowed to relax in a position of their own choice. The start and end of each trial were marked by an acoustic “beep”, and a 3-s verbal countdown was given before the start of each trial (30). After each trial, the total number of touches was noted down on the scoring sheet, and the best trial with the maximal number of touches was used for further analysis. The relative CKCUEST score was calculated as the mean number of touches divided by the subjects' body height (cm) while the CKCUEST power score was computed by multiplying the average number of touches by 68% of the subjects' body mass (kg) divided by 15 as proposed by Goldbeck and Davies (31) and Tucci et al. (32). For adolescents, the CKCUEST was reported as having a moderate to excellent reliability with an intersession reliability of the average touches score of ICC = 0.68, a relative score of ICC = 0.68, and a power score of ICC = 0.87 (33).

### Assessment of shoulder mobility/stability

A Y Balance Test Kit (Move2Perform, Evansville, IN) was used for each trial, and the values were noted down into an adapted Upper Quarter Y Balance (YBT-UQ) testing protocol. Prior to the trials, one experienced examiner demonstrated the correct execution of the test. Additionally, all subjects received a standardised verbal instruction before their first execution of the trials. All subjects started in a push-up position with their feet shoulder-wide apart (24) and the right arm being the first stance arm. Out of this position, the participants moved the indicator with their mobile hand into the medial (MD), inferolateral (IL), and superolateral (SL) directions. All three reach directions had to be performed consecutively without any break while maintaining the push-up position with the contralateral arm on the base of the device. The trial was invalid once the subject did not maintain the push-up position, pushed the indicator out of his reach (i.e., lost contact before the final position), or lost one of the three contact points on the floor (left and right foot and arm on the base of the device). The following trial started after a 30-s rest period with the same stance arm until all three trials were finished. After a 30-s rest period, the same procedure started with the left arm as the stance arm and the right arm as the mobile arm until also on this side all three trials were finished in the same manner. The values of all three directions were noted down for every trial, and the best score for each direction was taken for further analysis (24). Additionally, the composite score (CS) was calculated as the mean of the averaged maximal distances for all three reach directions, and all reaches were normalised for upper limb length. The intraclass correlation coefficient (ICC) of the YBT-UQ was reported to range from 0.91 to 0.95 in previous studies (34, 35).

### Assessment of throwing velocity

A 3 x 2 m target net (SG 500 L) was attached to a handball goal in the training venue of the participating teams. As an orientation for the subjects, the target net had a hole of a 1 m × 1 m in the middle of it as a fixpoint for the throws of the subjects. A “Stalker Pro” radar gun (Applied Concepts Inc., Richardson, TX, USA) was placed exactly behind this fixpoint and the respective goal net at a height of 1.20 m facing in the direction of the thrower to secure the Doppler effect. The “Stalker Pro” measures velocities ranging from 0 to 480 km/h with an accuracy of 0.16 km/h in a 0.01 s time interval with a working frequency of 35.1 GHz and a low disturbance threshold (36). One of the testers positioned himself behind the stalker to report the throwing velocity to the second tester who noted down the values on the scoring sheet of each subject. All players used the same standard ball size 3 including the same amount of glue. The contralateral leg of the subjects was placed behind the 7-m line facing the target net and holding the ball with their throwing arm. A bench was put alongside the 7-m line to assure that the subjects were blocked from moving forwards during or after the throwing attempts. All throws were executed as standing throws with no run-up, following the guidelines of the German Handball Federation (23) with a short rest between each throw. The highest throwing velocity of the three consecutive trials was noted down for further analysis. Only the throwing arm was tested. Throws with no run-up in this setting were reported to be highly reliable (ICC = 0.89) in a study by Rios et al. (37).

### Statistical analyses

Descriptive data are reported in terms of group mean values and standard deviations. A univariate analysis of variance (ANOVA) was performed to test for significant differences in participants' characteristics and pretest values between the two groups. Significant group differences occurred for the MD (*p* = 0.015) and the IL (*p* = 0.026) reach direction of the throwing arm as well as for MD (*p* = 0.026) reach direction of the non-throwing arm and were thus included as covariates in the statistical analyses. Thereafter, a series of univariate analysis of covariance (ANCOVA) was performed with the between-subject factor group (i.e., INT group, CON group) and including the aforementioned baseline measures as covariates. Muscular endurance, shoulder mobility/stability, and throwing velocity were used as dependent variables. Additionally, the partial eta squared ($\eta_{\text{p}}^{2}$) was used as an effect size measure and classified as small (0.02 ≤ $\eta_{\text{p}}^{2}$ ≤ 0.12), medium (0.13 ≤ $\eta_{\text{p}}^{2}$ ≤ 0.25), or large ($\eta_{\text{p}}^{2}$ ≥ 0.26). All statistical analyses were performed using Statistical Package for Social Sciences version 28.0 (IBM Corp., Armonk, NY, USA).

## Results

Table 3 displays statistics for all analysed variables. No injuries in relation to the executed training were suffered. The attendance rate was 96% in the INT group and 94% in the CON group.

**Table 3:** Intervention effects on measures of muscular endurance, shoulder mobility/stability, and athletic performance in male sub-elite adolescent handball players.

**Variables**	**INT–group (*n =* 13)**		**CON–group (*n =* 13)**		**ANCOVA statistics**	
	**Pre**	**Post**	**Pre**	**Post**	**Difference between means (95% CI)**	**p-value ($\eta_{\text{p}}^{2}$)**
**Muscular endurance**
Bourban test, ventral chain (s)	75.8 ± 26.1	104.0 ± 41.8	85.7 ± 43.1	65.9 ± 26.0	−38.1 (−9.9 to −66.3)	0.130 (0.09)
Bourban test, dorsal chain (s)	66.5 ± 25.3	89.3 ± 23.8	55.2 ± 17.6	60.6 ± 14.0	−28.7 (−12.9 to −44.5)	< 0.001 (0.46)
Bourban test, lateral chain, right side (s)	45.7 ± 20.5	60.9 ± 19.0	47.3 ± 20.9	39.6 ± 14.0	−21.3 (−7.8 to −34.8)	0.039 (0.17)
Bourban test, lateral chain, left side (s)	51.5 ± 19.8	64.9 ± 18.0	49.4 ± 25.2	39.7 ± 10.3	−25.2 (−13.3 to −37.1)	0.015 (0.22)
CKCUEST (best n)	29.9 ± 5.3	34.3 ± 2.8	29.5 ± 7.7	32.5 ± 4.5	−1.8 (1.2 to −4.9)	0.400 (0.03)
CKCUEST (mean n/height)	15.5 ± 2.9	18.5 ± 1.4	15.2 ± 3.7	16.8 ± 2.1	−1.7 (−0.3 to −3.1)	0.143 (0.09)
CKCUEST (power)	101.9 ± 19.8	122.5 ± 17.5	96.1 ± 34.1	105.5 ± 23.1	−17.0 (−0.4 to −33.6)	0.153 (0.08)
**Shoulder mobility/stability**
**Throwing arm reach**
MD (% AL)	116.7 ± 6.5	117.2 ± 10.6	108.6 ± 9.1	105.9 ± 7.7	−11.2 (−3.8 to −18.7)	0.095 (0.12)
IL(% AL)	111.9 ± 15.1	113.5 ± 11.9	100.2 ± 9.5	100.8 ± 11.8	−12.7 (−3.1 to −22.4)	0.221 (0.06)
SL(% AL)	82.5 ± 9.9	91.3 ± 8.6	85.0 ± 13.5	84.9 ± 13.7	−6.4 (2.8 to −15.7)	0.656 (0.01)
CS(% AL)	103.7 ± 8.8	107.3 ± 8.3	97.9 ± 8.8	97.2 ± 9.2	−1 0. 1 (−3. 0 to −1 7. 2)	0.024 (0.20)
**Non–throwing arm reach**
MD (% AL)	115.4 ± 8.2	116.1 ± 8.6	108.1 ± 7.5	107.7 ± 9.2	−8.4 (−1.2 to −15.6)	0.228 (0.06)
IL(% AL)	111.0 ± 14.1	111.2 ± 12.6	101.7 ± 11.0	99.5 ± 14.6	−11.8 (−0.8 to −22.8)	0.038 (0.17)
SL(% AL)	80.6 ± 11.7	91.2 ± 9.7	81.2 ± 12.7	81.1 ± 14.1	−10.1 (−0.3 to −19.9)	0.279 (0.05)
CS(% AL)	102.3 ± 8.9	106.2 ± 8.4	97.0 ± 9.2	96.1 ± 9.1	−1 0. 1 (−3. 0 to −1 7. 2)	0.027 (0.19)
**Athletic performance**
Throwing velocity (km/h)	76.4 ± 9.5	81.4 ± 8.4	76.5 ± 6.0	75.2 ± 8.2	−6.2 (0.5 to −13.0)	0.205 (0.07)

Values are mean values± standard deviations. Figures in brackets are effect sizes with 0.02 < $\eta_{\text{p}}^{2}$ < 0.12 indicating small, 0.13 < $\eta_{\text{p}}^{2}$ < 0.25 medium, 10 and $\eta_{\text{p}}^{2}$ > 0.26 large effects. AL, arm length; ANCOVA, analysis of covariance; CKCUEST, closed kinetic chain upper extremity stability test; CON, control group; CS, composite score; IL, inferolateral; INT, intervention group; MD, medial; SL, superolateral.

### Muscular endurance

**Figure 2:**
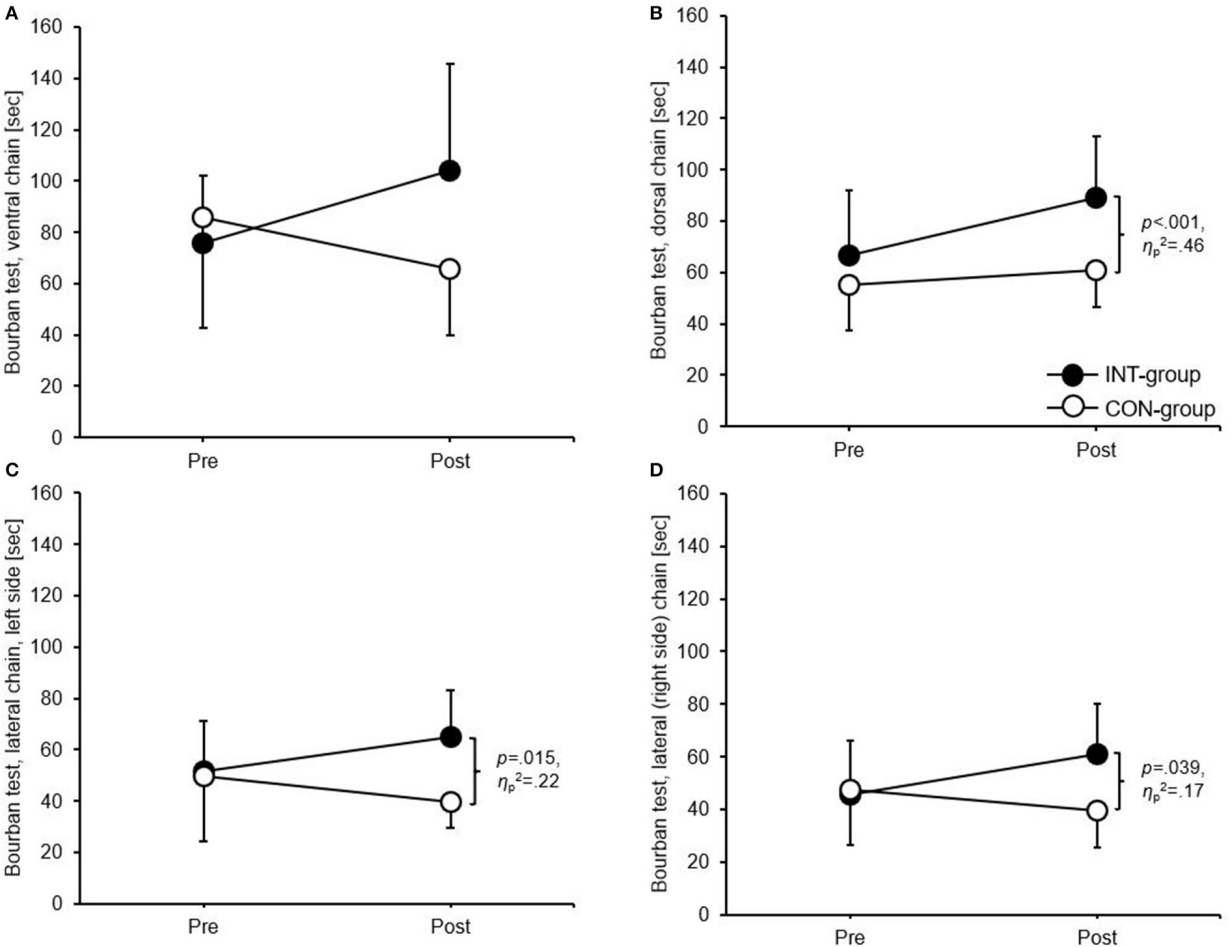
Group-specific intervention effects for the **(A)** ventral chain, **(B)** dorsal chain, **(C)** lateral (left side) chain, and **(D)** lateral (right side) chain of the Bourban test. Data are group mean values ± standard deviations. CON, control group; INT, intervention group.

For the Bourban test, the ANCOVA revealed significant differences between means in favour of the INT group for the dorsal chain (*p* < 0.001, $\eta_{\text{p}}^{2}$ = 0.46) and the lateral chain (left side: *p* = 0.015, $\eta_{\text{p}}^{2}$ = 0.22; right side: *p* = 0.039, $\eta_{\text{p}}^{2}$ = 0.17) (Figure 2). For the CKCUEST, no significant differences between means occurred.

### Shoulder mobility/stability

**Figure 3:**
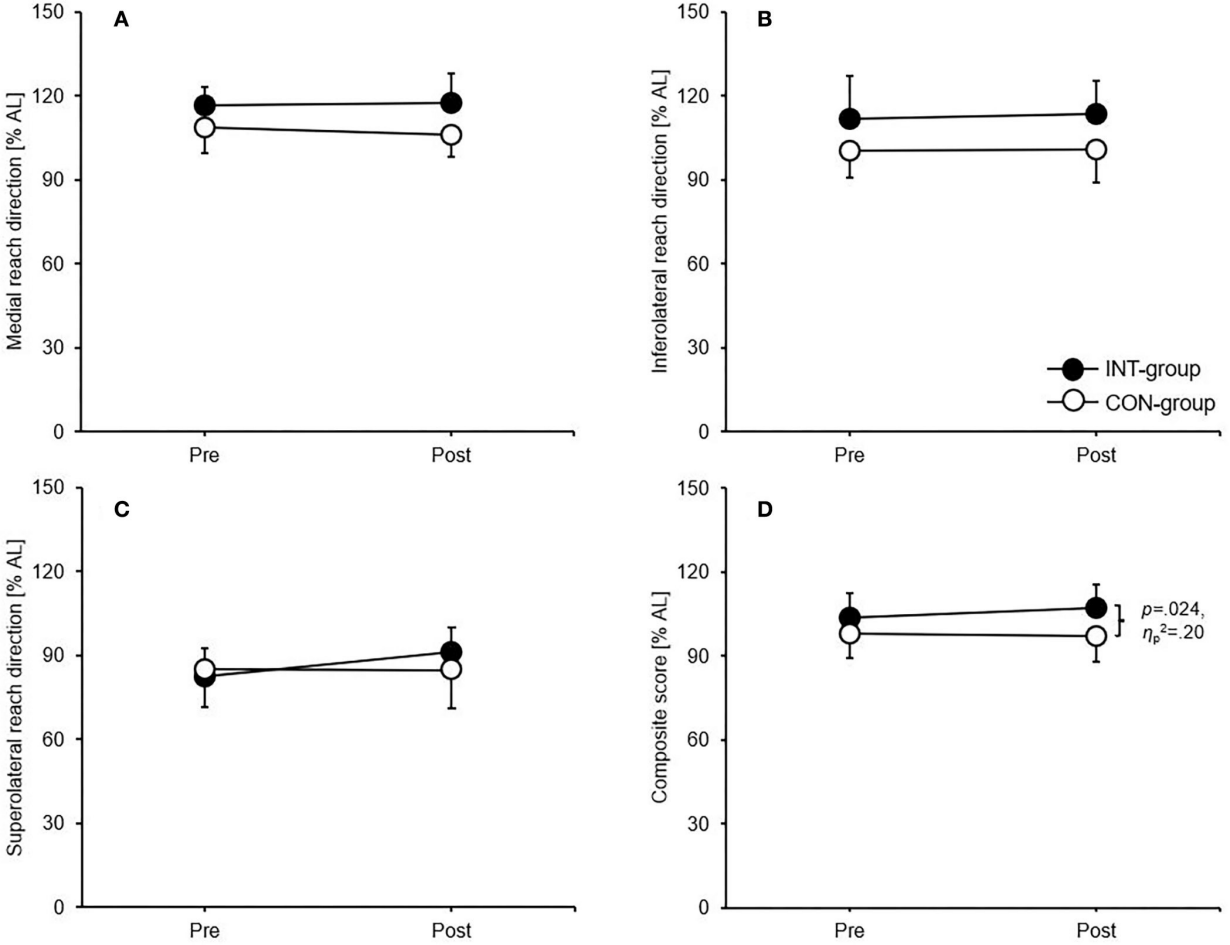
Group-specific intervention effects for the **(A)** medial reach direction, **(B)** inferolateral reach direction, **(C)** superolateral reach direction, and **(D)** composite score of the Upper Quarter Y Balance test (throwing arm reach). Data are group mean values ± standard deviations. AL, arm length; CON, control group; INT, intervention group.

**Figure 4:**
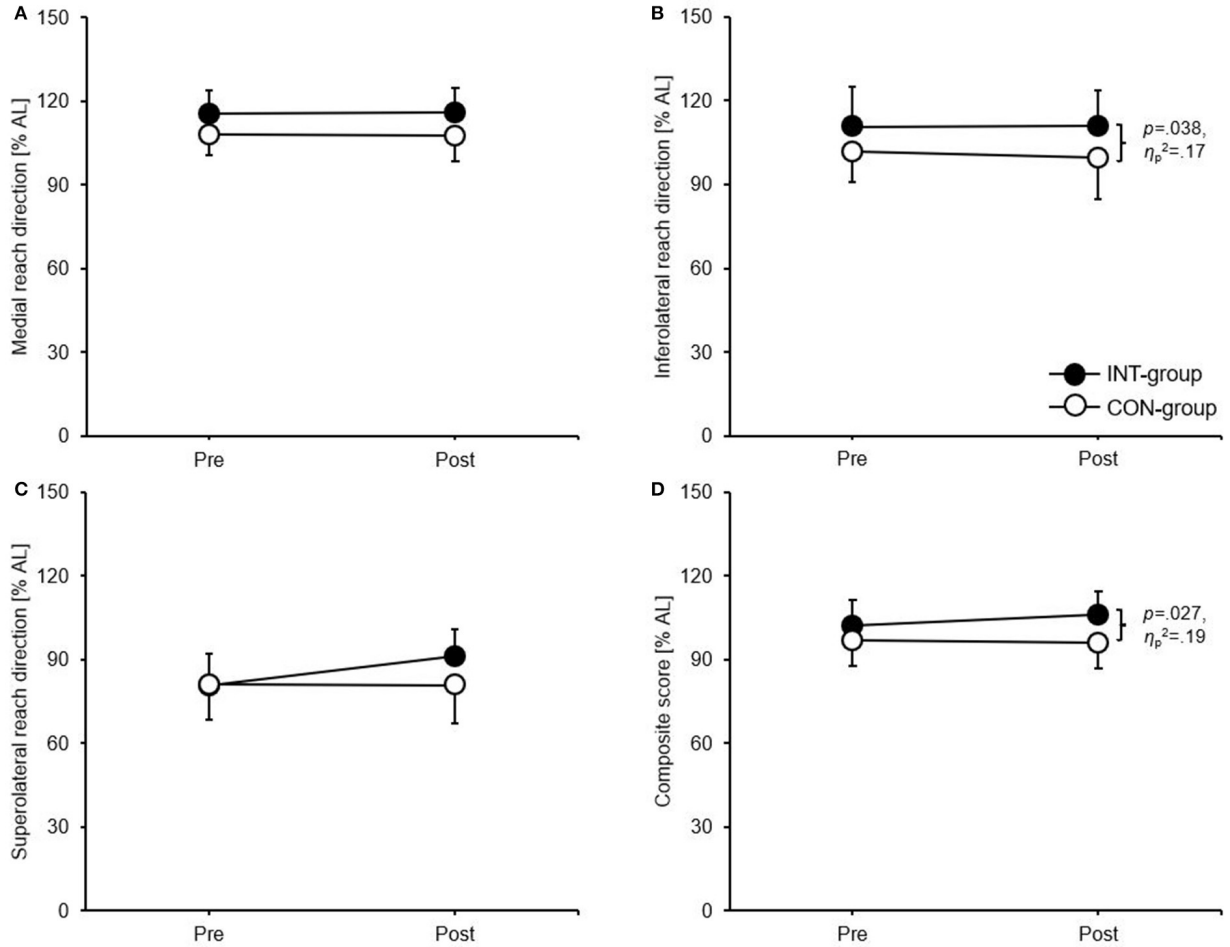
Group-specific intervention effects for the **(A)** medial reach direction, **(B)** inferolateral reach direction, **(C)** superolateral reach direction, and **(D)** composite score of the Upper Quarter Y Balance test (non-throwing arm reach). Data are group mean values ± standard deviations. AL, arm length; CON, control group; INT, intervention group.

For the throwing arm reach, the ANCOVA showed significant differences between means in favour of the INT group for the composite score (*p* = 0.024, $\eta_{\text{p}}^{2}$ = 0.20) (Figure 3). Concerning the non-throwing arm reach, significant differences between means in favour of the INT group occurred for the inferolateral reach direction (*p* = 0.038, $\eta_{\text{p}}^{2}$ = 0.17) and the composite score (*p* = 0.027, $\eta_{\text{p}}^{2}$ = 0.19) (Figure 4).

### Throwing velocity

The ANCOVA detected no significant differences between means.

## Discussion

The aim of the study was to investigate the effects of 6 weeks of core strengthening training on measures of physical (i.e., muscular endurance, shoulder mobility/stability) and athletic performance (i.e., throwing velocity) in adolescent male sub-elite handball players. The main results can be summarised as follows: a) significantly larger improvements in favour of the INT group were detected for measures of muscular endurance (i.e., dorsal and lateral left/right side chain of the Bourban test), shoulder mobility/stability (i.e., throwing arm reach: CS; non-throwing arm reach: IL reach direction and CS); b) for all other tests/measures, no significant group-specific changes from pre- to post-training were observed.

### Effects on measures of muscular endurance

We hypothesised greater improvements in muscular endurance in the INT compared to the CON group. Based on the present results, we can only partly confirm our hypothesis because only the dorsal and the lateral chains (left and right side) were significantly improved in favour of the INT group. In terms of the lateral chain, this improvement goes in line with Kuhn et al. (14) who also reported significant improvements in the lateral chain and a lack of improvements for the ventral chain also present in our study following a core strengthening training for 6 weeks. However, the significant improvements of the dorsal chain in our study were not present in the study by Kuhn et al. (14). The lack of significant improvements of the ventral chain may be due to the fact that during handball training, especially during throws, the ventral chain is highly trained (trunk flexion) leading to a lower adaptive reserve for improvements. Additionally, as stated by Wagner et al. (38), sport-specific training over several years limits the effect of an additional strength-oriented training twice a week. However, based on the reference values of Büsch et al. (27) the participants in the INT group improved in all reach directions from “below average” to “average”. Therefore, increasing the core strengthening training quantity to three sessions per week within the regular handball training seems to have an enhancing effect on muscular endurance as assessed by the Bourban test. Another reason for the significant findings of three out of the four chains may be that all chains profit from the “big 3” exercises as all of the exercises (i.e., the cross curl-ups, the side bridge, and the quadrupedal stance) lead to high forces at the core (39) with a high number of motor units being activated. This reasoning is supported by Cortell-Tormo et al. (40), Crommert et al. (41), and Oliva-Lozano and Muyor (42) who reported the highest electromyographic signals during curl-ups and in the plank position especially in the rectus abdominis and transversus abdominis as well as the quadratus lumborum and erector spinae as the antagonists at the back.

As far as the CKCUEST is concerned, the lack of significant differences may be due to the high baseline scores of the players (INT group: 29.9 ± 5.3 touches; CON group: 29.5 ± 7.7 touches) which consequently lead to a lower adaptive reserve (43). Additionally, the accompanying handball training with its endurance-oriented character and the repetitive throwing actions with its possible effects on the shoulder girdle may also have led to the lack of differences in the CKCUEST between groups as both executed nearly the same handball training.

### Effects on measures of shoulder mobility/stability

We further expected that both groups will improve their performances in shoulder mobility/stability with superior effects for the INT group. Again, our hypothesis can just partly be confirmed as only the CS of the throwing and the non-throwing arm reach and the IL reach of the non-throwing arm reach were significantly improved in favour of the INT group. This finding goes in line with possible correlations between shoulder mobility/stability and core strength in different populations (35, 44). As in our study partially improvements were detected for the parameters of muscular strength, positive adaptations in terms of shoulder mobility/stability as secondary effects of core strengthening training were also unlikely. The reason for the significant improvement of the IL reach direction of the non-throwing arm may be that the throwing arm as the stance arm is highly trained due to the repetitive throwing movements during training sessions therefore being able to hold up the one-arm push-up position for a long time (43). In this context, the subjects of the INT group demonstrated a rather good muscular endurance in the upper extremities as proved through the CKCUEST which although not being significantly improved from 29.9 ± 5.3 touches to 34.3 ± 2.8 touches following the intervention most likely enabled them to stabilise the stance arm for a longer time. The significant improvement of the IL direction during the non-throwing arm reach with the throwing arm as the stance arm may additionally be explained by the fact that this reach direction with the non-throwing arm as the mobile arm puts the highest demands on the core due to the rotational and translatory movement of the trunk. The improvements of the CS of both the throwing and the non-throwing arm reach may be the additive cumulative result of (nonsignificant) improvements of the three chains (except for the IL direction of the non-throwing arm reach) which were demonstrated in the improvements of all chains based on the classification of Büsch et al. (27).

The lack of improvements in the other reach directions may also be since the present core exercises are performed in a rather limited range of motion while the YBT-UQ is executed at a large range of motion at the end range of stability (34). In this line of thought, a recent review by Pallarés et al. (45) concluded that the improvement of functional performances appears to be favoured by higher range of movement exercises which were not executed in the present intervention.

### Effects on measures of athletic performance

In addition, we could not confirm our assumption that both groups will improve their handball-specific athletic performance (i.e., throwing velocity) and that the INT group will show larger enhancements as compared to the CON group. On the one hand, this goes in line with Kuhn et al. (14), Manchado et al. (16), and Ozmen et al. (18) who found no improvements in throwing velocity after core strengthening training interventions. On the other hand, the present finding contradicts Saeterbakken et al. (17) and Dahl and van den Tillaar (15) who did report improved throwing velocities following a core strengthening training. As a possible reason, the improvements in the lateral chains may not be specific enough for the improvement of throwing velocity as the throwing movement is mainly determined by trunk flexion, i.e., the ventral chain (8), which showed no significant improvements in our study. Therefore, improvement of lateral muscle endurance together with dorsal improvements may not sufficiently influence throwing velocity. Additionally, less motor control may be needed when performing the “big 3” core exercises compared with the highly complex skill of throwing with its numerous degrees of freedom (7). In this context, Clark et al. (46) criticised that isolated core strengthening training does not reflect the necessary full body core function that characterises dynamic athletic performance. In this regard, Saeterbakken et al. (17) and Dahl and van den Tillaar (15) used additional equipment (i.e., slings) during their exercises, probably enabling greater degrees of freedom which could additionally be responsible for the differences in the efficiency compared to our study. Moreover, our core strengthening exercises were performed lying on the floor while the handball-specific technique of throwing is executed in an upright position, representing a lack of the principle of training specificity in terms of position timing and functional specificity (47). Probably, more training sessions, i.e., >18 sessions, as proposed in the review of Saeterbakken et al. (12) would have led to better athletic performance values. Meta-analytical findings only indicated moderate effects for core strengthening training on sport-specific performance, small-to-large effects on physical fitness, and moderate effects on trunk muscle endurance (12).

### Limitations

There are some limitations that need to be addressed. The present results can only be transferred to adolescent male sub-elite players. Although core strengthening exercises are purported to improve the whole kinetic chain, the present results can mainly be transferred to the upper body as most assessments (e.g., YBT-UQ, CKCUEST) have a focus on it. Additionally, the fixed progression of core exercises on a team level may have led to different load of the exercises for everyone as progression is known to be rather on an individual than on a team level, and the present approach does not allow for individualised progression or load quantification. Concomitant with that, the randomisation protocol on a team rather than on an individual level is a further limitation. Probably, less trained athletes may have profited more from the present core strengthening training due to their higher adaptive reserve. Moreover, throwing technique was not analysed so probably core strengthening training may have led to a more economic transfer of force without improving throwing velocity. This may be especially true as only the standing throw was analysed. However, differences between the standing throw with no run-up, the penalty throw, the jumping throw, and the subsequent different kinetic chains have been reported (5). Additionally, core stability may refer to more static positioning (48) of the body while core strengthening training may require a more dynamic approach. As the ball can be considered a free weight, the inclusion of free weights in the training program may therefore be a more appropriate approach also for other athletic parameters that are executed in a dynamic upright position. Therefore, including exercises with more extensive movements of the extremities may lead to greater benefits in muscular endurance and/or mobility/stability of the upper extremities. An additional advantage of our core training program is that it does not need any materials and therefore can be easily organised.

## Conclusion

We investigated the effects of 6 weeks of core strengthening training on measures of physical (i.e., muscular endurance *via* Bourban test and CKCUEST, shoulder mobility/stability *via* YBT-UQ) and athletic performance (i.e., throwing velocity) in adolescent male sub-elite handball players. Our data suggest that a floor-based core strengthening training during the regular handball training sessions does lead to few improvements in physical but not athletic performance. Precisely, analyses demonstrated improvements in the dorsal and lateral left/right side chain of the Bourban test, the CS of the throwing arm reach as well as the IL reach direction and the CS of the non-throwing arm reach. Therefore, 6 weeks of core strengthening training were effective in improving some components of physical fitness but not in improving the functionally highly relevant handball-specific ability of throwing velocity. However, based on the relatively small investment of time and the fact that core strengthening is a feasible, safe, and easy to administer training mode, a specific core strengthening training may still be advantageous in terms of training stimulus variability and when a low load/low movement approach is sought especially following injuries and during rehabilitation periods.

## Data availability statement

The original contributions presented in the study are included in the article/supplementary material, further inquiries can be directed to the corresponding author/s.

## Ethics statement

The studies involving human participants were reviewed and approved by the Human Ethics Committee at the University of Duisburg-Essen, Faculty of Social Sciences approved the study protocol (TM_23.03.2020). Written informed consent to participate in this study was provided by the participants' legal guardian/next of kin.

## Author contributions

JB and TM designed the research question and analysed the data. JB planned and supervised the testing's, conducted the testing's and data collection, and wrote the main part of the manuscript. TM reviewed the manuscript. All authors approved the final manuscript.

## Funding

We acknowledge support from the Open Access Publication Fund of the University of Duisburg-Essen. The funding body is independent of the design of the study and collection, analysis, and interpretation of data and in writing the manuscript.

## Conflict of interest

The authors declare that the research was conducted in the absence of any commercial or financial relationships that could be construed as a potential conflict of interest.

## Publisher's note

All claims expressed in this article are solely those of the authors and do not necessarily represent those of their affiliated organizations, or those of the publisher, the editors and the reviewers. Any product that may be evaluated in this article, or claim that may be made by its manufacturer, is not guaranteed or endorsed by the publisher.

## Abbreviations

AL: arm length
ANOVA: analysis of variance
ANCOVA: analysis of covariance
BMI: body mass index
bpm: beats per minute
CKCUEST: Closed Kinetic Chain Upper Extremity Stability Test
CS: composite score
CON group: control group
IL: inferolateral
INT group: intervention group
MD: medial
SL: superolateral
YBT-UQ: Upper Quarter Y Balance test.
